# Nosocomial blood stream infection in intensive care units at Assiut University Hospitals (Upper Egypt) with special reference to extended spectrum β-lactamase producing organisms

**DOI:** 10.1186/1756-0500-2-76

**Published:** 2009-05-06

**Authors:** Shaaban H Ahmed, Enas A Daef, Mohammed S Badary, Mohammed A Mahmoud, Alaa A Abd-Elsayed

**Affiliations:** 1 Department of Microbiology and Immunology, Faculty of Medicine, Assiut University, Assiut, Egypt; 2 Department of Public Health and Biostatistics, Faculty of Medicine, Assiut University, Assiut, Egypt

## Abstract

**Aim:**

This study investigated the nosocomial blood stream infection (BSI) in the adult ICUs in Assiut university hospitals to evaluate the rate of infection in different ICUs, causative microorganisms, antimicrobial resistance, outcome of infection, risk factors, prevalence of extended spectrum B-lactamase producing organisms and molecular typing of *Klebsiella pneumoniae *strains to highlight the role of environment as a potential source of nosocomial BSI.

**Methods:**

This study was conducted over a period of 12 months from January 2006 to December 2006. All Patients admitted to the different adult ICUs were monitored daily by attending physicians for subsequent development of nosocomial BSI. Blood cultures were collected from suspected patients to detect the causative organisms. After antimicrobial susceptibility testing, detection of ESBLs was conducted among gram negative isolates. Klebsiella pneumoniae isolates were tested by PCR to determine the most common group of B-lactamase genes responsible for resistance. Klebsiella pneumoniae isolates from infected patients and those isolated from the environment were typed by RAPD technique to investigate the role of environment in transmission of infection.

**Results:**

The study included 2095 patients who were admitted to different ICUs at Assiut University Hospitals from January 2006 to December 2006. Blood samples were collected from infected patients for blood cultures. The colonies were identified and antibiotic sensitivities were performed. This study showed that the rate of nosocomial BSI was 75 per 1000 ICU admissions with the highest percentages in Trauma ICU (17%). Out of 159 patients with primary bloodstream infection, 61 patients died representing a crude mortality rate of 38%. Analysis of the organisms causing BSI showed that Gram positive organisms were reported in 69.1% (n = 121); MRSA was the most prevalent (18.9%), followed by methicillin resistant coagulase negative Staphylococci (16%). Gram negative bacilli were reported in 29.1% (n = 51). In this case, *Klebsiella pneumoniae *was the most common (10.3%) followed *E coli *(8.6%). *Candida spp*. was reported only in (1.7%) of isolates. Antibiotics sensitivities of Gram positive organisms showed that these organisms were mostly sensitive to vancomycin (90.1%), while Gram negative organisms were mostly sensitive to imipenem (90.2%). In this study we tested Gram negative isolates for the production of the ESBL enzyme and concluded that 64.7% (33/51) of patients' isolates and 20/135 (14.8%) environmental isolates were confirmed to be ESBL producers. The type of β-lactamase gene was determined by polymerase chain reaction which showed that SHV was the main type. Molecular typing was done for 18 Klebsiella pneumoniae strains that caused nosocomial BSI and for the 36 Klebsiella pneumoniae strains which were isolated from the environmental samples by the RAPD method. The two environmental strains were identical, with one isolated from a patient, which confirms the serious role of the hospital environment in the spread of infections.

**Conclusion:**

Nosocomial BSI represents a current problem in Assiut University Hospitals, Egypt. Problems associated with BSI include infection with multidrug resistant pathogens (especially ESBLs) which are difficult to treat and are associated with increased mortality. Of all available anti-microbial agents, carbapenems are the most active and reliable treatment options for infections caused by ESBL isolates. However, overuse of carbapenems may lead to resistance of other Gram-negative organisms.

## Findings

Nosocomial infections (NIs) are infections that became clinically evident after 48 hours of hospitalization and do not originate from patient's original admitting diagnosis [1]. These infections cause significant morbidity and mortality and have a considerable impact on healthcare costs. Among all types of NIs, the nosocomial BSI creates a serious health problem in hospitals all over the world. In addition, patients admitted to ICUs carry an even higher risk of nosocomial BSI than those admitted to other types of units. Data from the surveillance and control of pathogens of epidemiologic importance (SCOPE) surveillance system in United States hospitals showed that 49.4% of all nosocomial BSI occurred in the ICU [2]. Reports on the incidence of BSI vary significantly, reflecting differences in individual risk based on institution, co-morbid illnesses, length of stay, and hospital location. Moreover, on a national level, nosocomial BSI is the 10^th ^leading cause of death in the U.S. Approximately 250,000 cases of BSI occur in the U.S. annually [3].

In the last 30 years, the frequency, etiology, and epidemiology of nosocomial BSI have changed with the evolution of medical care, particularly among the increasing number of hospitalized patients who require intensive care. Nearly 75% of primary bloodstream infections have been caused by Gram-negative bacilli. This is due to the development of potent anti-staphylococcal β-lactam agents. *Staphylococcus aureus *gave way to Gram-negative bacilli, however, by the early 1980s; Gram-positive cocci began to re-emerge as predominant nosocomial pathogens [4].

Mortality and morbidity from infections are greater when caused by antimicrobial-resistant bacteria. Enterobacteriaceae and non-fermentative Gram-negative bacilli are of great concern because antimicrobial therapy for infections due to these resistant pathogens remains a clinical dilemma in hospitalized patients [5]. It is also noted that there is an increase in the resistance among Gram-negative bacilli to third generation cephalosporins which is caused by expression of Extended-Spectrum B-lactamase (ESBL) enzymes. Therefore infections due to ESBL isolates continue to pose a challenge to infection management world wide [6].

This study aimed at investigating the nosocomila BSI in the adult intensive care units in Assiut university hospitals to evaluate the rate of infection in different ICUs, causative microorganisms, antimicrobial resistance, outcome of infection, prevalence of extended spectrum B-lactamase producing organisms and molecular typing of *Klebsiella pneumoniae *strains to highlight the role of the environment as a potential source of nosocomial BSI.

## Methods

### Study Design and Subjects

This study was approved by the ethical committee of our institute and written informed consent was obtained from all patients for publication of this manuscript.

#### Patients

The study was conducted over a period of 12 months from January 2006 to December 2006 to all patients admitted at different adult ICUs at Assiut University Hospitals including: Coronary care unit (CCU), Chest ICU, Tropical ICU, Neurological ICU, Internal Medicine ICU, General ICU, Trauma ICU and Neurosurgical ICU. All patients admitted at different adult intensive care units (ICUs) were monitored daily by attending physicians for subsequent development of nosocomial BSI, which must meet at least one of the following criteria:

Criterion 1: Patient has a recognized pathogen cultured from one or more blood cultures and the organism cultured from blood is not related to an infection at another site.

Criterion 2: Patient has at least one of the following signs or symptoms: fever (> 38°C), chills, or hypotension and at least one of the following:

Common skin contaminant (e.g., diphtheroids, Bacillus sp., coagulase-negative staphylococci, or micrococci) is cultured from two or more blood cultures drawn on separate occasions. The Signs and symptoms of infection appear 48 hours to four days after admission, and there are no signs or symptoms of infection at the time of admission, proven by history and clinical examination [7].

Controls: included 159 Patients admitted to different ICUs during the same period but without any signs or symptoms of infection. Each case was matched with one control for age, sex and ICU specialty.

Environmental assessment: 475 environmental samples were collected from walls, floors, beds, bedside tables, and trays. These are the sites that we usually sample from based on the protocol followed in our institute.

Contact precautions were taken into consideration for the medical staff, hospital workers and visitors.

### Sample collection

Blood samples: 5 to 10 ml of venous blood was collected from patients using sterile syringes. Blood samples were inoculated immediately under complete aseptic conditions into bottles containing 50 ml of brain heart infusion broth [8].

Environmental samples: sterile cotton swabs were moisten by a sterile physiological saline solution and used to collect samples from walls, floors, beds, bedside tables, and trays.

### Processing of samples

Blood samples: the blood culture bottles were incubated aerobically at 37°C for 7 days. The bottles were examined daily for evidence of bacterial growth as haemolysis, gas production or turbidity above the red cell line. Subcultures using sterile syringes were done on blood agar, chocolate agar, MacConkey's agar and Bile Esculin Azide agar daily for 7 days before reporting blood cultures as negative [9]. Isolation of anaerobes is not considered.

Environmental samples: they were inoculated aerobically at 37°C for 24 hours on brain heart infusion broth for enrichment and then sub-cultured on blood agar and incubated for 24 hours at 37°C.

### Identification of bacterial isolates

Identification of gram negative bacilli: gram negative bacilli were identified by API 20E system (Biomeriux SA, Montalien Vercica and France).

Identification of enterococcal isolates: suspected enterococcal isolates on Bile Esculin Azide agar were identified by the Colony morphology, Gram staining, the catalase test, the PYR test (production of pyrrolidonyl arylamidase), and the possession of Lancefield antigen D by using commercially available latex agglutination test. Omega Avipath Strep (Omega Diagnostica LTD., Scotland, UK).

Identification of staphylococcal isolates: according to ***Louie et al***. [10], staphylococci were identified by standard methods including the gram stain, catalase test and tube coagulase test. Samples were cultured on Mannitol Salt Agar (MSA) (Oxoid, UK), where S. aureus produces yellow colonies (1 mm in diameter) surrounded by a yellow medium, and CNS forms small orange colonies surrounded by a red or purple medium [11].

Isolates which showed positive growth on Mannitol Salt Agar plates were subcultured on an Oxacillin Resistant Screening Agar Base (ORSAB) medium (Oxoid, UK) for Detection of oxacillin resistance.

### Antimicrobial Susceptibility testing by modified Kirby-Bauer disc diffusion method: [10]

According to the protocol of the infection control lab, the following antibiotics were used: Antibiotics for Gram positive bacteria: B-Lactams (Ampicillin, Penicillin G, Cefazoline, Amoxicillin/clavulanic acid, Ceftriaxone, Cefotaxime and Oxacillin), Macrolides (Erythromycin), Aminoglycosides (Amikacin, Gentamicin), Tetracyclines (Tetracycline), Quinolones (Ciprofloxacin, Norfloxacin), Others (Chloramphenicol, Clindamycin, Vancomycin, Teicoplanin, Trimethoprim/sulfamethoxazole)

Antibiotics for Gram negative bacteria: B-Lactams (Ampicillin, Penicillin G, Cefazoline, Amoxicillin/clavulanic acid, Ceftriaxone, Ceftazidime, Piperacillin, Cefoperazone, Cefpodoxime, Cefuroxime, Aztreonam, Imipenem) Aminoglycosides (Amikacin, Gentamicin, Tobramycin), Tetracyclines (Tetracycline), Quinolones (Ciprofloxacin, Norfloxacin), Others (Chloramphenicol, Trimethoprim/sulfamethoxazole).

### Detection of β-lactamase production

β-lactamase enzyme is detected among the gram negative bacilli by the chromogenic method (Nitrocefin test).

### Detection of Extended Spectrum B-Lactamases

Selective testing for ESBL production is considered for all gram negative bacilli.

Screening for ESBL-Production: [12].

Isolates that exhibited reduced susceptibility to one or more of cefpodoxime, ceftazidime, aztreonam, cefotaxime or ceftriaxone were considered as potential producers of ESBL.

Confirmatory Tests:

The combined disk method or Inhibitor potentiated disc diffusion test [12]: ceftazidime (30 μg) versus ceftazidime/clavulanic acid (30 μg/10 μg), (Oxoid, UK), were used as a phenotypic confirmatory test where a greater than or equal to 5 mm increase in the zone diameter for the antimicrobial agent tested in combination with B-lactamase inhibitor versus its zone when tested alone indicates ESBL production.

Double-Disk Synergy "DDS" Test [13]: where any enhancement in the zone of inhibition between a beta-lactam disk and one containing the beta lactamase inhibitor was indicative of the presence of an ESBL.

ESBL – E – Test [14]: According to the manufacturer, a ceftazidime MIC/ceftazidime clavulanic acid MIC ratio which is equal to or greater than 8 indicates the presence of ESBLs (positive test).

### Determination of the type of β-lactamase by polymerase chain reaction

In the present study, 18 *Klebsiella pneumoniae *strains which have caused primary bloodstream infection were investigated to determine the probable type of β-lactamase enzyme which was responsible for resistance. The isolates were picked up and tested for TEM, SHV, CTX-M-1, TOHO-1 genes by the PCR method [15].

### Molecular typing by Random Amplified Polymorphic DNA (RAPD)

18 *Klebsiella pneumoniae *strains isolated from nosocomial BSI cases and 36 *Klebsiella pneumoniae *strains isolated from the environmental samples were typed by the RPAD method.

RAPD is a method for detecting strain differences. Its ability to type a wide variety of bacterial strains in a short time suggests that it will be a useful molecular epidemiological tool [16].

### Statistical analysis

**Figure 1:**
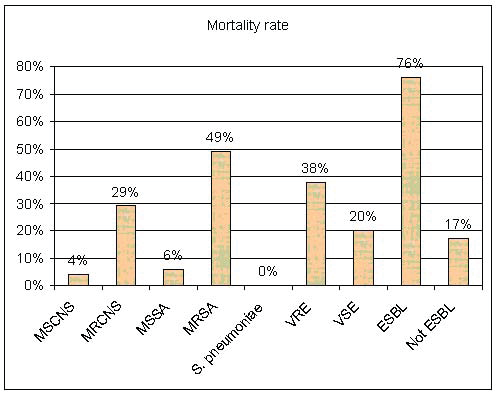
**Variation in mortality rate by the organism which causes primary bloodstream infection**.

Data entry and statistical analysis was done using the SPSS ver.15 which included descriptive analysis, logistic regression for calculation of risk factors and dendrogram analysis. Figure 1 was done by using the MS excel 2003 program.

### Consent

Written informed consent was obtained from the patients for publication of this manuscript.

## Results

### Analysis of nosocomial BSI

From January to December 2006, 2095 patients were admitted to different ICUs at Assiut University Hospitals. 17.5% of those patients developed nosocomial infections. Among these nosocomial infections, nosocomial BSI was the most common, accounting for 43% (n = 159). The rate of nosocomila BSI was 44 per 1000 patient days.

The rate of bacteremia among different ICUs is illustrated in table 1, which shows that the highest percentages were in trauma ICU (17%) followed by coronary care unit (13%) and general ICU 9.3%.

**Table 1:** Rate of bacteremia among different ICUs

**ICU**	**Frequency**	Percent (%)*
Trauma (n = 171)	29	17
Post operative (n = 100)	3	3
Chest (n = 375)	31	8.3
Medicine (n = 159)	6	3.8
General (n = 225)	21	9.3
Neurological (n = 190)	11	5.8
Coronary (n = 331)	43	13
Tropical (n = 260)	9	3.5
Neurosurgical (n = 284)	6	2.1
Total (n = 2095)	159	7.6

*(Percentage is calculated according to the number of patients admitted to each ICU)

### Microorganisms isolated from patients with BSI

Out of 159 bloodstream infections, 175 microorganisms were isolated. Ninty percent (143/159) of infections were monomicrobial, while (16/159) 10% of infections were poly-microbial, containing 2 microorganisms. These microorganisms and their distribution among different ICUs are shown in table 2.

**Table 2:** Isolated microorganisms and their distribution among different ICUs.*

Organism	ICU									Total No and % Of the organism in all ICUs (n = 175)
		
	Trauma n = 33	Post operative n = 4	Chest n = 30	Medicine n = 6	General n = 25	Neurological n = 12	Coronary n = 48	Tropical n = 10	Neurosurgical n = 6	
MS CNS	2(6.1%)	1(25%)	3(100%)	1(16.7%)	3 (12%)	4(33.3%)	9(18.8%)	2(20%)	0(0%)	25(14.3%)
MR CNS	6(18.2%)	1(25%)	2(*6.7%)	1(16.7%)	7(28%)	0(0%)	9(18.8%)	0(0%)	2(33.3%)	28(16%)
MSSA	3(9.1%)	0(0%)	5(16.7%)	2(33.3%)	2(8%)	0(0%)	3(6.3%)	1(10%)	2(33.3%)	18(10.3%)
MRSA	10(30.3%)	0(0%)	8(26.7%)	2(33.3%)	4(16%)	3(25%)	2(4.2%)	3(30%)	1(16.7%)	33(18.9%)
Streptococcus pneumoniae	0(0%)	0(0%)	0(0%)	0(0%)	0(0%)	0(0%)	4(8.3%)	0(0%)	0(0%)	4(2.3%)
Enterococcus spp.	1(3%)	0(0%)	2(6.7%)	0(0%)	4(16%)	1(8.3%)	5(10.4%)	0(0%)	0(0%)	13(7.4%)
E coli	2(6.1%)	1(25%)	3(10%)	0(0%)	3(12%)	0(0%)	5(10.4%)	0(0%)	1(16.7%)	15(8.6%)
Klebsiella pneumoniae	4(12.1%)	0(0%)	4(13.3%)	0(0%)	1(4%)	2(16.7%)	5(10.4%)	2(20%)	0(0%)	18(10.3%)
Klebsiella oxytoca	0(0%)	0(0%)	0(0%)	0(0%)	0(0%)	0(0%)	0(0%)	1(10%)	0(0%)	1(0.6%)
Enterobacter spp.	2(6.1%)	0(0%)	2(6.7%)	0(0%)	1(4%)	2(16.7%)	3(6.3%)	1(10%)	0(0%)	11(6.3%)
Acinetobacter baumannii	1(3%)	0(0%)	0(0%)	1(16.7%)	0(0%)	0(0%)	2(4.2%)	0(0%)	0(0%)	4(2.3%)
Pseudomonas aeruginosa	0(0%)	0(0%)	1(3.3%)	0(0%)	0(0%)	0(0%)	1(2.1%)	0(0%)	0(0%)	2(1.1%)
Candida spp.	2(6.1%)	1(25%)	0(0%)	0(0%)	0(0%)	0(0%)	0(0%)	0(0%)	0(0%)	3(1.7%)

*(n = total number of isolates in the ICU, percentage is calculated per number of isolates in each ICU)

Analysis of these isolates showed that Gram positive organisms were reported in 69.1% (n = 121); MRSA was the most prevalent (33/175, 18.9%), followed by methicillin resistant coagulase negative Staphylococci (16%). Gram negative bacilli were reported in (51/175, 29.1%). *Klebsiella pneumoniae *was the most common (28/175, 10.3%) followed E coli (8.6%). Candida spp. was reported only in (1.7%) of isolates.

### Crude Mortality

Out of 159 patients with nosocomial BSI, 61 patients died, representing a crude in-hospital mortality rate of 38% with a great variation in mortality rate by organism (figure 1). The predisposing risk factors for BSI are summarized in tables 3, which show that nosocomial BSI was significantly associated with previous use of antibiotics, diabetes, leucocytopenia, surgery, central intravascular catheters and mechanical ventilation.

**Table 3:** Odds ratio, 95% confidence intervals, and P values for variables in logistic regression analysis of nosocomial BSI.

*Risk factor*	*P value*	*OR*	*95.0% C.I.*	
			
			Lower	Upper
Previous administration of antibiotics	0.002	7.43	3.34	9.542
Diabetes	0.001	1.050	1.010	1.751
Obesity	0.154	1.43	0.328	2.868
Male nutrition	0.528	1.353	0.529	3.459
Immune deficiency	0.287	2.562	0.454	14.470
Leucocytopenia	0.01	3.756	2.361	5.028
Surgery	0.005	6.702	2.882	12.283
Urinary Catheter	0.066	1.669	0.967	2.882
Peripheral I.V. Catheter	0.094	1.547	0.269	2.109
Central I.V. Catheter	0.013	5.654	3.184	8.821
Assisted nutrition	0.09	4.961	2.790	8.818
Mechanical Ventilation	0.027	4.833	3.959	9.506
Endotracheal tube	0.171	1.692	0.797	3.595
Drainage tube	0.128	1.122	0.026	2.571
Renal dialysis	0.538	1.471	0.043	3.158

### Antimicrobial Susceptibility testing

Antibiotics sensitivities of gram positive organisms showed that the isolated organisms were mostly sensitive to vancomycin (90.1%), amikacin (50.4%) and chloramphenicol (43.8%), however gram negative organisms were mostly sensitive to imipenem (90.2%), amikacin (45.1%) and gentamicin (33.3%).

### Analysis of Environmental Samples

Analysis of these samples showed that 210/475 (44.2%) samples were clean and 265/475 (55.8%) were contaminated with different microorganisms. The distribution of these microorganisms appears in table 4, which shows that the highest contamination rates were in Trauma ICU (82%) followed by Chest ICU (77.8%) and Post operative ICU 36 (75%).

**Table 4:** Distribution of pathogens isolated from the environment among different ICUs

***Organism***	***ICU***									***Total n = 475***
		
	**Trauma n = 67**	**Post operative n = 48**	**Chest n = 54**	**Medicine n = 53**	**General n = 50**	**Neurological n = 51**	**Coronary n = 50**	**Tropical n = 52**	**Neurosurgical n = 50**	
**MS CNS**	9(13.4%)	2(4.2%)	5(9.3%)	1(1.9%)	3(6%)	3(5.9%)	1(2%)	0(.0%)	4(8%)	28(5.9%)
**MR CNS**	4(6%)	0(.0%)	6(11.1%)	6(11.3%)	4(8%)	2(3.9%)	3(6%)	2(3.8%)	0(.0%)	27(5.7%)
**MSSA**	12(18%)	20(41.7%)	2(3.7%)	5(9.4%)	0(.0%)	3(5.9%)	0(.0%)	0(.0%)	0(.0%)	42(8.8%)
**MRSA**	0(.0%)	1(2.1%)	6(11.1%)	1(1.9%)	2(4%)	4(7.8%)	3(6%)	2(3.8%)	5(10%)	24(5.1%)
***Enterococcus spp***.	1(1.5%)	2(4.2%)	1(1.9%)	0(.0%)	0(.0%)	1(2%)	0(.0%)	0(.0%)	0(.0%)	5(1.1%)
***E coli***	16(23.9%)	9(18.8%)	1(1.9%)	1(1.9%)	1(2%)	3(5.9%)	1(2%)	0(.0%)	0(.0%)	32(6.7%)
***Klebsiella pneumoniae***	4(6%)	2(4.2%)	15(27.8%)	2(5.7%)	1(2%)	5(9.8%)	3(6%)	0(.0%)	4(8%)	36(7.6%)
***Pseudomonas aeruginosa***	5(7.5%)	0(.0%)	3(5.6%)	2(3.8%)	3(6%)	2(3.9%)	1(2%)	0(.0%)	1(2%)	17(3.6%)
***Candida spp***.	0(.0%)	0(.0%)	0(.0%)	3(5.7%)	0(.0%)	0(.0%)	0(.0%)	1(1.9%)	0(.0%)	4(0.8%)
**Nonlactose fermenter**	0(.0%)	0(.0%)	3(5.6%)	10(19%)	14(28%)	0(.0%)	0(.0%)	0(.0%)	1(2%)	28(5.9%)
***Proteus spp***.	4(6%)	0(.0%)	0(.0%)	0(.0%)	0(.0%)	6(11.8%)	9(18%)	3(5.9%)	0(.0%)	22(4.6%)
**Contamination rate**	55(82%)	36(75%)	42(77.8%)	31(58.4%)	28(56%)	29(56.8%)	21(42%)	8(15.3%)	15(30%)	265(55.8%)

*(n = number of samples obtained from each ICU, percentage is calculated according to number of samples obtained from each ICU

### Analysis of β-lactamase producers

Out of the 175 organisms which caused BSI, 29.1% (51/175) were Gram negative bacilli. All these strains were tested for the production of the β-lactamase enzyme by the nitrocefin test, which indicated that 82.4% (42/51) were β-lactamase producers.

### Analysis of ESBL production

Preliminary screening showed that 78.4% (40/51) of patients' isolates and 24.4% (33/135) of the environmental isolates were considered as potential producers of ESBLs.

Confirmatory tests (Combined Disk method, Double-Disk Synergy "DDS" Tests, and ESBL E-Test) were carried out to confirm the production of ESBL. Taking all confirmatory tests into account, a total of 64.7% (33/51) of patients' isolates and 14.8% (20/135) of the environmental isolates were confirmed to be ESBL producers.

### Determination of the type of β-lactamase by PCR

18 *Klebsiella pneumoniae *strains were isolated from patients with nosocomial bacteremia and analyzed by PCR. Our aim was to determine the probable type of β-lactamase gene which is responsible for resistance.

It was found that SHV was the main type of β-lactamase (61.1%). TEM was second (55.6%), CTX-M1 (38.9%) was third and there was no TOHO-1 group of β-lactamases. Some Klebisella pneumoniae strains produced more than one type of β-lactamase. Two strains produced both enzymes (TEM and SHV, 11.1%); one strain produced TEM and CTX-M-1 enzymes (5.6%) and six strains produced TEM, SHV and CTX-M-1 enzymes (33.3%).

### Molecular typing by Random Amplified Polymorphic DNA (RAPD)

**Figure 2:**
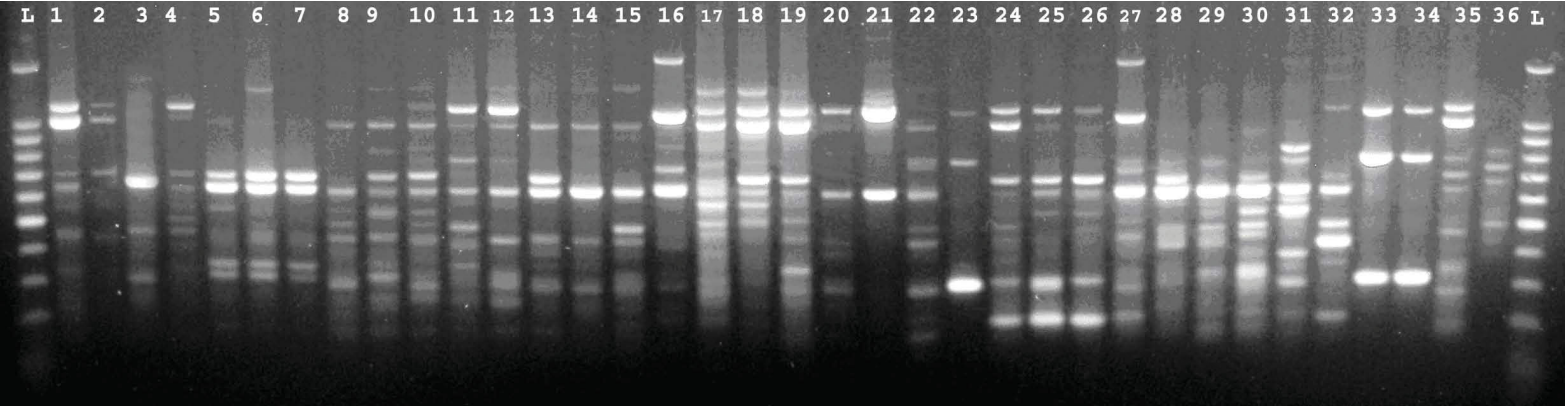
**RAPD patterns of: 36 *K. pneumoniae *isolated from the environment**.

**Figure 3:**
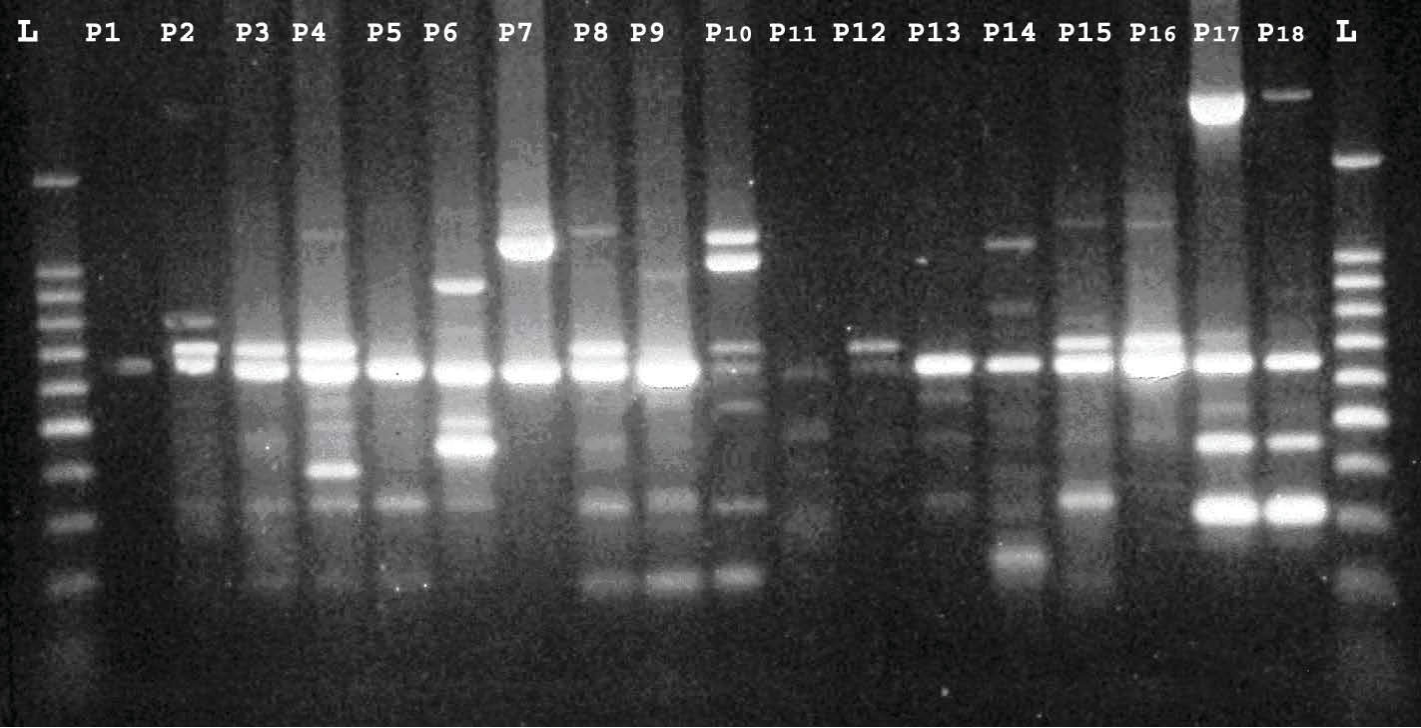
**RAPD patterns of: *18 *strains isolated from patients**.

-The PCR conditions allowed the amplification of 1–13 bands ranging in size from 220–2200 bp (figure 2, 3). The strains were considered to be identical if they differed by one band.

**Figure 4:**
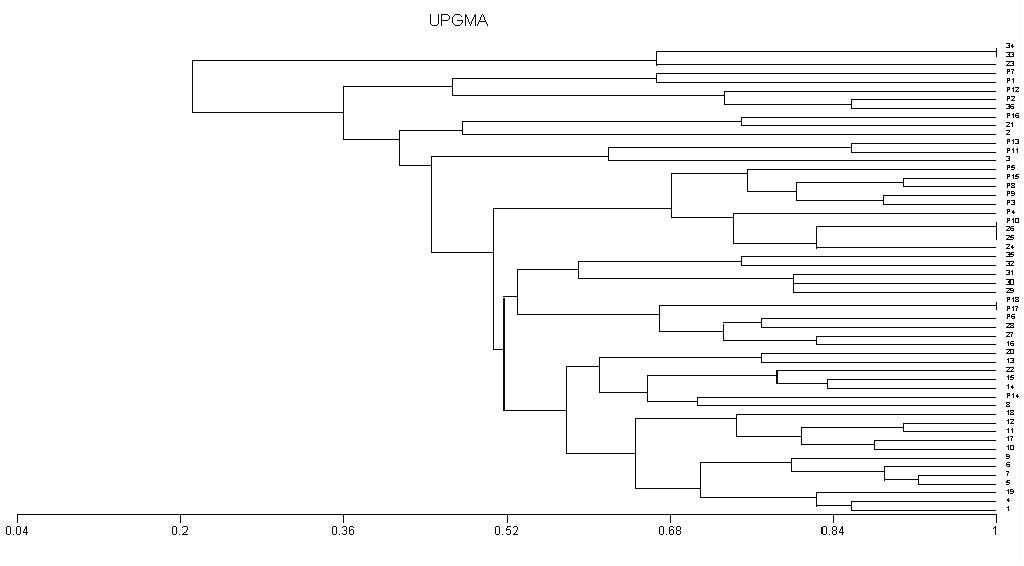
**The corresponding dendrogram**. **1→36 **= environmental samples, ***P1*→*P18 ***= patient samples

The RAPD pattern of the patient samples P17 and P18 were identical (figure 4).

The RAPD pattern of the environmental sample 33 is identical with that of environmental sample 34 (figure 4).

In addition, one RAPD group – environmental samples 25 and 26 with patient sample P10, showed three identical patterns. By referring to the clinical data of these samples, it was found that these samples were isolated from Trauma ICU and occurred in the same time period. (figure 4).

These identical RAPD patterns suggest that the corresponding strains constitute a **clonal lineage **[17].

## Discussion

In the present study, the rate of nosocomial BSI per 1000 patient days was 44. These results were higher than those reported by ***Sligl et al***.[18] in Canada. The highest number of cases was reported form the coronary ICU (43/159 patients).

In this study we tried to assess the risk factors associated with BSI. Higher BSIs were found among diabetic patients with a statistically significant difference, which can be explained by the low immunity of diabetic patient and their high vulnerability to infections.

In addition, there was a tendency towards increasing the percentage of BSI among patients with previous prescriptions of antibiotics and mechanical ventilation. This is supported by findings of ***Sǔljagic et al***.[2] who reported that risk factors for acquisition of BSI in the ICU included previous prescription of antibiotics, mechanical ventilation and the use of nasogastric tubes. In conclusion, multivariate analysis demonstrated that previous administration of antibiotics; diabetes, central intravenous lines, leucocytopenia, and surgery were independently associated with BSI.

Identification of the microorganisms causing BSI showed that Gram positive organisms accounted for 69% of isolates, while Gram negative isolates accounted for 29% of them. We found a shift to Gram positive organisms as reported in many countries.

The leading pathogens causing clinically significant nosocomial BSI in our institution were coagulase negative staphylococci *(30.3%), S. aureus *(29.2%) and *Klebsiella pneumoniae *(10.3%). Our results are consistent with the results of other surveillance studies. Data from the SCOPE (Surveillance and Control of Pathogens of Epidemiologic Importance) project revealed that the most common pathogens causing nosocomial BSI were coagulase-negative staphylococci (32%) and S. aureus (16%) [19]. In addition, data published from the National Nosocomial Infections Surveillance System (NNIS) for ICU-associated primary bloodstream infections identified coagulase-negative staphylococci (37%) and S. aureus (12.6%) as the leading pathogens [20]. Other studies by ***Warran et al***. [21] also confirmed the emergence of Gram-positive pathogens in nosocomial BSI. This increase in the frequency of coagulase-negative staphylococcal BSI isolates has occurred concurrently with the increased use of invasive intravascular catheters. In addition, the trend for coagulase negative staphylococci may reflect a change from regarding these organisms as skin flora to viewing them as clinically significant.

Gram negative pathogens were lower on the rank of organisms and included *Klebsiella pneumoniae *(10.3%) which was the most commonly isolated Gram negative organism, followed by *E coli *(8.6%) and *Enterobacter spp*. (6.3%). These results are similar to those of other recent nosocomial BSI studies where these organisms have been among the leading Gram-negative pathogens [3]. Antibiotic resistance is a growing problem in hospitals everywhere.

In the present study, 65% of the *S. aureus *blood-stream isolates are resistant to methicillin. The incidence of methicillin resistance was also high among coagulase-negative staphylococci, with 53% of CNS being resistant to methicillin. Our finding coincides with that reported in a French general hospital, where the MRSA rate was 46% [22].

Gram negative rods showed high resistance rates to the majority of antibiotics. This may be explained by the late introduction of infection control programs in our hospital, the site where our study was performed (ICUs only) and the high percentage of extended spectrum β lactamases among Gram negative bacilli (64.7%), which limits the therapeutic choices in infections caused by such strains due to two broad factors: cross-resistance (e.g. to aminoglycosides, co-trimoxazole or fluoroquinolones) and the spectrum of these enzymes.

In our study, the crude in-hospital mortality rate associated with nosocomial BSI was 38%. Similarly, in a nationwide study, a crude mortality rate as high as 27% has been reported [3].

In addition, the present study identified a great variation of crude mortality rate by pathogen. Higher rates were obtained with MRCNS (29%) compared to MSCNS (4%), with MRSA (49%) compared to MSSA (6%), with VRE (38%) compared to VSE (20%) and with ESBLs (76%) compared to Non ESBLs (17%). Also, investigators in Canada have compared the mortality between MRSA and MSSA bacteremia and reported a higher mortality in the MRSA group (36 vs. 20%), but this failed to achieve statistical significance [23]. Furthermore, the mortality of MRSA bacteremia was 11.8% versus 5.1% in MSSA with no statistical significance. These data suggest that the mortality of MRSA bacteremia exceeds that of bacteremia with MSSA.

Analysis of environmental samples showed that 265 samples (55.8%) were contaminated with different microorganisms. The most common contaminating organisms were MSSA 42 (8.8%), followed by *K. pneumoniae *36(7.6%) and *E. coli *32 (6.7%). The highest contamination rate occurred in Trauma ICU (82%), followed by Chest ICU (77.8%) and Post operative ICU (75%).

Correlation between nosocomial BSI rate and environmental analysis showed that the highest rate of BSI was in Trauma ICU (17%). These results highlighted the role of the environment in the spread of nosocomial BSI and agreed with ***Karanfil et al***. [24] who found that the inanimate contaminated environment which induces surfaces and surgical instruments is a potential source of nosocomial BSI. Detection of ESBL production was done by preliminary screening that depends on reduced susceptibility to one or more of cefpodoxime, ceftazidime, aztreonam, cefotaxime or ceftriaxone. This showed that 78.4% of patients' isolates and 24.4% of the environmental isolates were considered as potential producers of ESBLs.

Taking all confirmatory tests into account, a total of 64.7% of patients' isolates and 14.8% of environmental isolates were confirmed to be ESBL producers. Analysis of the patients' isolates showed that similar results were obtained by the Combined Disk method, the Double-Disk Synergy "DDS" test with clavulanic acid and the ESBL E-Test, with 26 isolates being found to be ESBL producers. The double disk synergy test using tazobactam revealed an additional 7 strains that were positive for ESBL. This suggests that those 7 strains produce an ESBL enzyme and a AmpC-enzyme. With these organisms, clavulanic acid may induce expression of high-level AmpC production and may then antagonize rather than protect the antibacterial activity of the partner β-lactam [25]. Thus, the presence of an ESBL can be masked by the expression of an AmpC-type enzyme in the same strain (by masking any synergy with ESBLs) [26]. As a result, when testing for ESBL production in these organisms, we should consider using sulfones such as tazobactam and sulbactam as β-lactamase inhibitors [27]. Although the exact reasons are not completely understood, the pressure to use non-β-lactam antibiotics may be responsible for the co-existence of ESBL and AmpC. The genes coding for ESBLs and other non-β-lactam resistance genes (e.g. aminoglycosides or co-trimoxazole) often reside within the same conjugative plasmid and therefore are transmitted together from one strain to another and can be co-selected under the pressure of prior multi-drug usage [28].

In our study, the percentage of extended spectrum β-lactamase producing Gram negative bacilli among patients with BSI was 64.7%. The prevalence and relative distribution of ESBLs vary depending on the facility and the level of care taken to control nosocomial BSI. These factors also vary with geographic location and time [29].

During our study, 14.8% of environmental Gram-negative isolates were ESBL-producers. These organisms are considered a potential source of primary bloodstream infection with ESBL-producing organisms which have limited therapeutic options, are multidrug resistant, and lead to poor outcomes [30]. The distribution of environmental ESBL-producing organisms showed that the highest rate was in the Coronary care unit (12%) followed by Trauma ICU (7.5%). Also, the highest rates of nosocomial BSI with ESBL-producing organisms were in the Coronary care unit (3.3%) and Trauma ICU (2.9%). These results confirm the serious role of the hospital environment in the transmission and spread of infections.

In the present study, the type of β-lactamase gene was determined among *Klebisella pneumoniae *strains by using a polymerase chain reaction, which showed that SHV was the main type of β-lactamase, followed by TEM and CTX-M1. There was no TOHO-1 group of β-lactamases. Some *Klebisella pneumoniae *strains produced more than one type of β-lactamase; TEM and SHV were produced by 2 isolates, while TEM and CTX-M-1 were produced by one isolate. Moreover, six strains produced TEM, SHV and CTX-M-1 enzymes.

SHV was the main type of β-lactamase, and TEM was second. Although we could not determine the subtype of TEM or SHV, these results at least suggest that TEM or SHV-type ESBLs probably exist. Similar results were reported by ***Bradford ***[26] who observed that TEM- and SHV-type ESBLs remain more common in North America.

In this work, we investigated unrelated strains from both the environment and infected patients. We found 50 RAPD genotypes among 54 *K. pneumoniae *studied, which demonstrated the high discriminatory power of RAPD with primer usage.

Two RAPD groups showed two identical patterns.

The clonal similarity between two patients in the same chest unit who were admitted at the same time and showed the same antibiogram suggests that this strain was transmitted from one patient to the other (may be the role of contaminated instruments or contaminated hands of hospital staff). Similar findings were reported by ***Lopesa et al***. [17] who found 4 strains isolated from an outbreak with *K. pneumoniae *to be related by RAPD analysis, suggesting clonal linearity.

Those two strains were isolated from the chest ICU Environmental samples and occurred in the same time period which indicates the importance of implying infection control measures on cleaning surfaces.

One of the interesting findings in this study is that 1 RAPD group, environmental samples 25 and 26 with the patient sample P10, showed 3 identical patterns. By referring to the clinical data of these samples, it was found that these samples were isolated from the Trauma ICU and occurred in the same time period. The environmental samples could have been transmitted to the patient, or they may have been a focus of infection to other patients which are highly susceptible due to different risk factors and the ICU setting.

The RAPD analysis indicated that pathogenic *K. Pneumoniae *strains comprise a genetically high variable group of organisms. These data confirmed the observation of ***Lai et al***. [30], who stated that based on the distribution of different nucleotide sequences; the pathogenic *K. Pneumoniae *population was highly heterogeneous.

In summary, *K. pneumoniae *infections have been caused by a variety of strain genotypes that could be transmitted from one patient to another in different ways, and it is important to monitor such strains closely to prevent their spread. In this situation, the design of rational infection control measures that require the adoption of new antibiotic policies in addition to improving hospital hygiene becomes even more challenging [17].

### Limitations of the study

The large number of patients did not align with the limited number of investigators. As a result, we recommend the development of a continuous data base registration system for acquired infection data. This will markedly help investigators in the future by allowing them to have access to a large set of data over many years. It will also help the detection of the effects of currently active hospital control measures.

## Conclusion

Nosocomial BSI represents a current problem in Assiut University Hospitals. Problems associated with BSI include infection with multidrug resistant pathogens (especially ESBLs) which are difficult to treat and are associated with increased mortality. Carbapenems are the most active and reliable treatment options for infections caused by ESBL isolates, however, overuse of carbapenems may lead to resistance of other gram-negative organisms.

## Competing interests

The authors declare that they have no competing interests.

## Authors' contributions

SA, ED and MB carried out the patient diagnosis, investigation, management and follow-up. MM, AAA-E carried out the patient diagnosis, investigation, management, follow-up, general coordination, drafting of the manuscript and writing the final manuscript. All authors have read and approved the final manuscript.
